# Supplementary material for: Assessing genome-wide dynamic changes in enhancer activity during early mESC differentiation by FAIRE-STARR-seq
Source: Nucleic Acids Res. 2021 Nov 24;49(21):12178–95. doi: 10.1093/nar/gkab1100 (PMC8643627; doi:10.1093/nar/gkab1100)
Supplement: gkab1100_Supplemental_Files [file gkab1100_supplemental_files.zip › LG_STARR_mESCs_Suppl_info_rev.pdf]

SUPPLEMENTARY DATA

Assessing genome-wide dynamic changes in enhancer activity during early mESC differentiation by FAIRE-STARR-seq

Laura V. Glaser[1*], Mara Steiger[1], Alisa Fuchs[1,2], Alena van Bömmel[1] , Edda Einfeldt[1], Ho-Ryun Chung[1,3], Martin Vingron[1], Sebastiaan H. Meijsing[1,4]

[1] Department of Computational Molecular Biology, Max Planck Institute for Molecular Genetics, 14195 Berlin, Germany

[2] The Berlin Institute for Medical Systems Biology, Max Delbrück Center for Molecular Medicine, 10115 Berlin, Germany

[3] Institute for Medical Bioinformatics and Biostatistics, Philipps University of Marburg, 35037 Marburg, Germany

[4] Max Planck Unit for the Science of Pathogens, 10117 Berlin, Germany

* Corresponding author: glaser@molgen.mpg.de

Content:

- Supplementary materials and methods

- Supplementary figures

- References

Supplementary materials and methods

FAIRE-STARR-seq

To asses enhancer activity, E14 cells were transfected with this plasmid library using a Nucleofector™ 2b device using the Mouse ES Cell Nucleofector Kit (Lonza, VAPH-1001). For each of the three f replicates, four individual transfections, each with 5 µg plasmid library and $5x10^6$ cells, were performed. The medium was changed 12 h after transfection and to half of the cells either LIF or 1 µM RA was added. After an additional 4 h of incubation, samples were pooled and RNA was isolated using the RNeasy Midi kit (Qiagen). Poly adenylated RNA was enriched using Dynabeads™ Oligo$(dT)_{25}$ (Invitrogen), residual DNA was digested using Turbo DNase (Invitrogen), and finally RNA was cleaned-up with Agencourt® RNAClean® XP beads (Beckman Coulter). cDNA was synthesized using SuperScript™ III Reverse Transcriptase (Invitrogen) according to the manufacturer's protocol, applying a reporter transcript-specific primer. This primer contains the sequence of the Illumina PCR Primer 2.0 as overhang as well as eight random nucleotides that serve as unique-molecular identifiers (UMI) for each cDNA molecule (CAAGCAGAAGACGGCATACGAGAT$[N]_8$GTGACTGGAGTTCAGACGTGTGCTCTTCCGATCT). cDNA was further amplified as described in Arnold *et al.* (1), using adjusted reporter-specific primers based on Illumina's TruSeq dual index system (universal: CAAGCAGAAGACGGCATACGA, sample specific: AATGATACGGCGACCACCGAGATCTACAC[barcode, n=6]ACACTCTTTCCCTACACGACGCTC).

As input control for FAIRE-STARR-seq, the input plasmid library was sequenced as well. To this end, the plasmid library was used for a pseudo "cDNA synthesis", using the random-UMI primer and the KAPA HiFi HotStart ReadyMix (Roche) for 4 cycles with a prolonged synthesis step (70 sec) to individually label input fragments. In a second step, this input library was amplified with Illumina's TruSeq dual index based universal and barcoded primers, as done for the FAIRE-STARR-seq libraries, using the KAPA HiFi HotStart ReadyMix (Roche) for 12 PCR cycles.

Library validation

To determine the complexity of the FAIRE fragments, we sequenced the plasmid input library (Fig. 1A) resulting in the identification of 4.4 million individual fragments, which cover about 186,000 significantly enriched open regions. As expected, the enrichment of our input regions resembles chromatin accessibility determined by DNaseI- or ATAC-seq at these sites (Fig. 1B, S1E). Accordingly, correlation analyses of genome-wide read distribution further confirmed a high correlation of our input library with DNaseI- and ATAC-seq profiles (Fig. 1C and S1F), validating that our library captured open regions, which are enriched for regulatory elements (2), on a genome-wide scale.

This plasmid input control we used for normalization does not undergo the exact same procedure as a plasmid extraction control after transfection and therefore may not be completely equivalent. However, we frequently observed very high correlation between input and recovered input libraries (3).

Assessment of interferon response upon transfection of mESCs with the FAIRE-STARR library

Upregulation of interferon genes in response to transfection with plasmids can also distort STARR-seq reporter activation (4). To test if this is a potential problem in the mESCs used in our study, we analyzed the expression levels of selected interferon response associated genes. However, for each of the genes analyzed, the levels were below the qPCR detection limit regardless of whether the cells were transfected or not (data not shown) indicating that the interferon response is not activated upon transfection and thus should not influence the STARR activity read-out in our assays. This is in line with published reports of a lacking type I interferon response in mESCs (5).

ChIP-seq

For ChIP experiments, E14 cells were washed once with PBS, treated with trypsin (Sigma, T4049) for 5 min and gently but thoroughly resuspended in ES medium to generate single cell suspensions. Cells were diluted to $20x10^6$ cells/20 ml medium and crosslinked by adding formaldehyde (1% v/v) for 5 min under gentle rotation. The reaction was quenched by adding 125 mM Glycine for an additional 5 min, then cells were washed three times with PBS, snap frozen in liquid nitrogen, and stored at -80°C.

HM ChIP experiments were preformed according to the standard BLUEPRINT protocol (www.blueprint-epigenome.eu): Cells were resuspended in shearing buffer (20 mM Tris pH 7.5, 150 mM NaCl, 2 mM EDTA, 1% Triton X-100, 0.1% SDS) supplemented with Complete Protease Inhibitor Cocktail (PIC) EDTA-free (Roche, 11873580001) and sheared on a Bioruptor Pico device for 25-35 cycles. For each ChIP, 1 µg antibody (listed in Table S2) was used. Automatic ChIP was performed using the SX-8G Compact IP-Star liquid handler (Diagenode) in combination with Auto Histone ChIP kits (Diagenode, C01010022). Using the pre-programmed method 'indirect ChIP', ChIP reactions were carried out in a final volume of 200 µl for 10 h followed by 5 h incubation with protein A magnetic beads and 5 min washes at 4°C. After the ChIP, eluates were recovered, RNase A-treated, de-crosslinked overnight at 65°C and treated with Proteinase K for 4 h at 55°C. The recovered DNA was purified using the ChIP DNA Clean & Concentrator Kit (Zymo research, D5205). Sequencing libraries were prepared using the NEBNext Ultra DNA Library Prep kit (NEB, E7370) according to manufacturer's instructions and submitted for paired-end Illumina sequencing on the HiSeq 2500.

The RARα ChIP was performed as described elsewhere (6), with the following modifications: Cells were cross-linked for 5 min with 1% formaldehyde and a mild sonication buffer was used (20 mM Tris-HCl pH 8.0, 2 mM EDTA pH 8.0, 1% Triton X-100, 150 mM NaCl, 0.1% SDS, 1x PIC). Prior to sonication, nuclei were incubated for 20 min on ice and homogenized ten times by a 27G needle. Per ChIP 4 µl RARα antibody (serum, Diagenode C15310155) or 2 µg IgG control (Diagenode C15410296) was used. Sequencing libraries for RARα ChIP and Input fragments were prepared using the KAPA Hyper Prep Kit (Roche) and submitted for paired-end Illumina sequencing on the NovaSeq 6000 generating 50 bp reads.

NGS data analyses

FAIRE-STARR-seq data analyses

FAIRE-STARR-seq libraries were sequenced with a HiSeq 2500 (Illumina) to generate 50 bp paired-end reads. Sequencing reads were aligned to the mouse genome (mm9) using Bowtie2 (7)(-X 800 --fr --very-sensitive). UMI-tools (8) was used for UMI-aware removal of PCR duplicates. SAMtools (9) was used to filter reads for proper pairs, alignment and quality scores (-h -b -f 3 -F 780 -q 5), to select reads mapping only to regular chromosomes (chr1-19, chrX and chrY), and to remove reads mapping to blacklisted regions (ENCFF547MET). UMI-aware deduplication of reads removed about 90% of obtained reads (Fig. S1B) and is aimed at retaining only true independent transcript replicates of one enhancer resulting in an overall decrease in read-counts for individual fragments (Fig. S1C). Genome-wide correlation analyses of read distributions of individual FAIRE-STARR-seq samples showed higher correlation coefficients when UMI-aware removal of read duplicates was omitted since overamplified fragments with very high read counts in two compared samples result in overestimation of correlation of read distributions. Fragments with extremely high read counts in only one replicate are prevalent without UMI-aware removal of duplicates, whereas these regions are absent after UMI-aware deduplication analysis (Fig. S1D) indicating that such regions are PCR amplification artefacts. Accessible regions covered by the input library were identified using MACS2 (10)(-q 0.05 --keep-dup all --call-summits -bw 200). Significantly active enhancers, using the input library as control, were called using MACS2 (10). The analysis was performed for each biological STARR-seq replicate individually as well as for the merged reads from all replicates. Finally, peaks were only counted as active STARR-seq enhancers when they were called for the merged reads and for at least two of three biological replicates and are covered by at least three individual fragments. Normalized STARR-seq

signal for data visualization was generated using bamCoverage of the deepTools package (11) for the replicate-merged STARR-seq reads or the input library to normalize for genomic coverage and sequencing depth (-of bigwig -bs 10 -e --normalizeUsing RPGC --effectiveGenomeSize 2304947926 --pseudocount 1). Next, signal tracks were normalized to input library coverage using bigwigCompare (-of bigwig -bs 10 --operation subtract --pseudocount 1). Genome browser snapshots depict FAIRE-STARR signal normalized to RPGC only, unless indicated otherwise. Heatmaps which show STARR-seq signal (RPGC and input normalized) distribution at selected regions were generated using computeMatrix (reference-point mode) and plotHeatmap tools of the deepTools package (11). Genomic distribution of FAIRE-STARR with respect to RefSeq genes was annotated with ChIPSeeker (12).

In order to score the FAIRE-STARR-seq enhancers, the computeMatrix tool of the deepTools package (11) was used, this time to obtain the average enhancer activity signal (input and read depth normalized tracks by bigwigCompare, see above (--operation log2)) over the size-scaled regions (scale-regions mode). Thus, the STARR score corresponds to log2((RNA from STARR-seq)/(DNA from input library)) per enhancer element. Clustering of FAIRE-STARR enhancers by enrichment of HMs was performed using the computeMatrix tool (scale-region mode to average HM enrichment per region) and k-means clustering (k was estimated by the elbow method (total within-cluster sum of square)). Subsequently, distributions of HMs, TFs, accessibility by ATAC, promoter annotation (RefSeq), transcription (RNA-seq), and enhancer prediction probability by CRUP (13) were plotted for the clustered regions with computeMatrix (reference-point mode on summit of the clustered regions) and plotHeatmap (11).

Correlation analyses

Genome-wide correlation analyses for read distributions were performed using multiBamSummary (deepTools (11)) and filtered reads. The genome was binned into 100 bp bins, fragments per bin were counted (bins -e -bs 100), the resulting table was analyzed in R (14) and pair-wise Pearson correlation coefficients and coefficients of determination were calculated.

ChIP-seq analyses

Paired-end ChIP-seq reads were mapped to the reference genome (mm9) using Bowtie2 (7)(--sensitive), and if applicable, mapped reads from the same experiment but different sequencing runs were merged. SAMtools (9) was used to filter for proper pairs, alignment and quality scores (-h -b -f 3 -F 780 -q 10), to select reads mapping only to regular chromosomes (chr1-19, chrX and chrY), and to remove reads mapping to blacklisted regions (ENCFF547MET). Input and sequencing depth normalized signal tracks were computed with bamCompare (-of bigwig --operation subtract -bs 25 --smoothLength 50 -e --normalizeUsing RPKM --ignoreDuplicates) (11). Significant RARα binding sites over input sample were identified using MACS2 (10). For RARα enhancer inducibility analysis, only RARα binding sites which overlap with the FAIRE-STARR input library (6,528 of 11,366 RARα sites) were included.

Reprocessing of deposited NGS data

If signal tracks were not available, NGS data for experiments listed in Table S2 were downloaded via fastq-dump, mapped to mm9 reference genome using Bowtie2 (7)(--sensitive), and if applicable, mapped reads from the same experiments but different sequencing runs were first merged and then filtered (-h -b -f 3 -F 780 -q 3) with SAMtools (9). Signal tracks were computed with bamCoverage or bamCompare (-of bigwig (--operation subtract) -bs 25 --smoothLength 50 -e --normalizeUsing RPKM --ignoreDuplicates) (11) depending on the availability of a control sample (indicated in Table S2). Reads mapping to blacklisted regions (15) were excluded. For deposited signal tracks mapped to mm10 reference genome, lift-over to mm9 was performed using CrossMap (16).

RNA-seq analysis

50 bp paired-end sequencing reads were aligned to the mouse genome (mm9) using STAR (17)(version 2.5.3a) and ENSEMBL genes (NCBIM37) as annotation reference. SAMtools (9) was used to filter reads for proper pairs, alignment and quality scores (-h -b -f 3 -F 780 -q 10), to select reads mapping only to regular chromosomes (chr1-19, chrX and chrY), and to remove reads mapping to blacklisted regions (ENCFF547MET). Fragments per gene were assessed using featureCounts (18) and ENSEMBL gene annotation. To compare expression between different groups of genes of the same treatment, transcripts per million reads (TPM) were calculated and compared. Normalization of read coverage and differential gene expression analysis for different treatments were performed using DESeq2 and LCF shrinkage (19). To compare and plot mean expression of genes between different treatments, TMM-normalized counts (20) were calculated with the edgeR package (21). To generate signal tracks for plotting RPKM normalized read coverage at example loci or heatmaps, bamCoverage was used (-of bigwig -bs 10 -e --normalizeUsing RPKM)(11).

ATAC-seq analysis

50 bp paired-end sequencing reads were aligned to the mouse genome (mm9) and filtered as described for ChIP-seq analysis. Signal tracks for plotting normalized read coverage at example loci or heatmaps were generated applying bamCoverage (-of bigwig -bs 25 --smoothLength 50 -e --normalizeUsing RPGC --effectiveGenomeSize 2304947926 --ignoreDuplicates)(11).

Motif enrichment analyses

To identify TF motifs enriched in sequences of interest, AME (22) was applied (--scoring avg --method fisher --hit-lo-fraction 0.25 --evalue-report-threshold 79 --control --shuffle--) using the JASPAR 2018 clustered vertebrate motif database (23) as input motifs. Results were analyzed in R (14), filtered by E-value thresholds as indicated, and plotted with the ggplot2 package (24). The JASPAR 2018 vertebrate core motifs and their corresponding clusters are listed in Table S4. To investigate the enrichment of RARα::RXRα motifs with different spacer lengths and half-site orientations, the corresponding scoring matrices were created by combining the monomers of the RARα::RXRα consensus motif (MA0159.1) into direct, inverted, and everted repeats with zero to eight nucleotides spacing. For the spacers, a uniform nucleotide frequency distribution was inserted to generate maximal degeneracy.
Counting of enriched motifs per fragment was performed using the matrix-scan function of the pattern matching program from RSAT software suite (25) with a first-order Markov model estimated from the input sequences as a background model and applying a p-value cut-off (0.002) to the predicted binding sites.

Heatmaps and anchor plots

Heatmaps and anchorplots depicting ChIP-, DNase-, ATAC-, or RNA-seq distribution or mean enrichment at selected genomic regions respectively, were generated using computeMatrix (reference-point mode) and subsequently plotHeatmap or plotProfile tools of the deepTools package (11). Sequencing depth and, if applicable and available, input normalized signal tracks were used.

Assignment of genes to enhancers and gene ontology analysis

To assign putative target genes to STARR enhancers we applied GREAT version 3.0.0 (26) using the whole genome (mm9) as background regions and for association setting "basal plus extension" with proximal: 5 kb upstream and 1 kb downstream, plus distal: up to 100 kb. The expression levels of assigned genes per enhancer group or cluster was plotted as TPM derived from RNA-seq. Additionally, GREAT performs a gene ontology analysis per analyzed enhancer group and provides enriched GO-

terms and significance levels, which were analyzed and cutoffs determined in R (14) and subsequently plotted with the ggplot2 package (24).

Classifier for enhancer and E-promoter prediction

Pre-processing and motif enrichment: As outlined in Fig. 4A, the 186,959 significantly enriched regions of the FAIRE-STARR input library were first divided into regions which do (16,769) or do not (170,190) overlap with ENSEMBL (NCBIM37) promoters, which were defined as regions of -500 bp to the TSS, and subsequently used to train an E-promoter and enhancer classifier, respectively. For each group, regions were ranked for their STARR activity (Fig. 4B and S4A) and the sequences of the highest and lowest ranking 10 or 1% for E-promoters or enhancers, respectively, were used for training of the classifier. The motifcounter tool (27) was used with default options to calculate sequence-wise motif enrichment of the 79 clustered motifs from JASPAR matrix clustering 2018 (23) using the union from both sets as background model. Since the width of highest and lowest STARR-scoring regions was significantly different (Wilcoxon $p < 1e-50$), region-width was included as a feature of the classifier. Negative log-transformed p-values of motif enrichment were generated and all variables were scaled such that they have the same mean and standard deviation, in order to allow for inferences about feature importance directly from regression model coefficients.

Fitting and evaluation of classifier: To differentiate between the highest and lowest ranking enhancers based on enrichment of the clustered TF motifs and motif width, a logistic regression model with elastic net regularization was built. The model combines ridge and lasso penalties to obtain shrunken and grouped coefficients, that prevent the regression model from overfitting (28). For training and evaluation of the model, a nested cross-validation approach was performed, where the inner loop is used for the optimization of hyperparameter λ (regularization penalty) and the outer loop to assess the predictive performance on unseen data. Additionally, the second hyperparameter α was tested over a grid of various values to find the optimal mixing percentage of lasso and ridge regression. Since only marginal differences in performance were observed, a value of $\alpha = 0$ corresponding to ridge regression was chosen to include enrichment of each of the clustered motifs in the classifier. Model performance for each of the outer cross-validation folds was assessed via the receiver operating characteristic (ROC) curve to derive a mean and standard deviation of the AU-ROC (area under the ROC curve). Preprocessing, training, and testing of the model were performed with R using the glmnet package (29) for elastic-net regularized models.

Supplementary figures

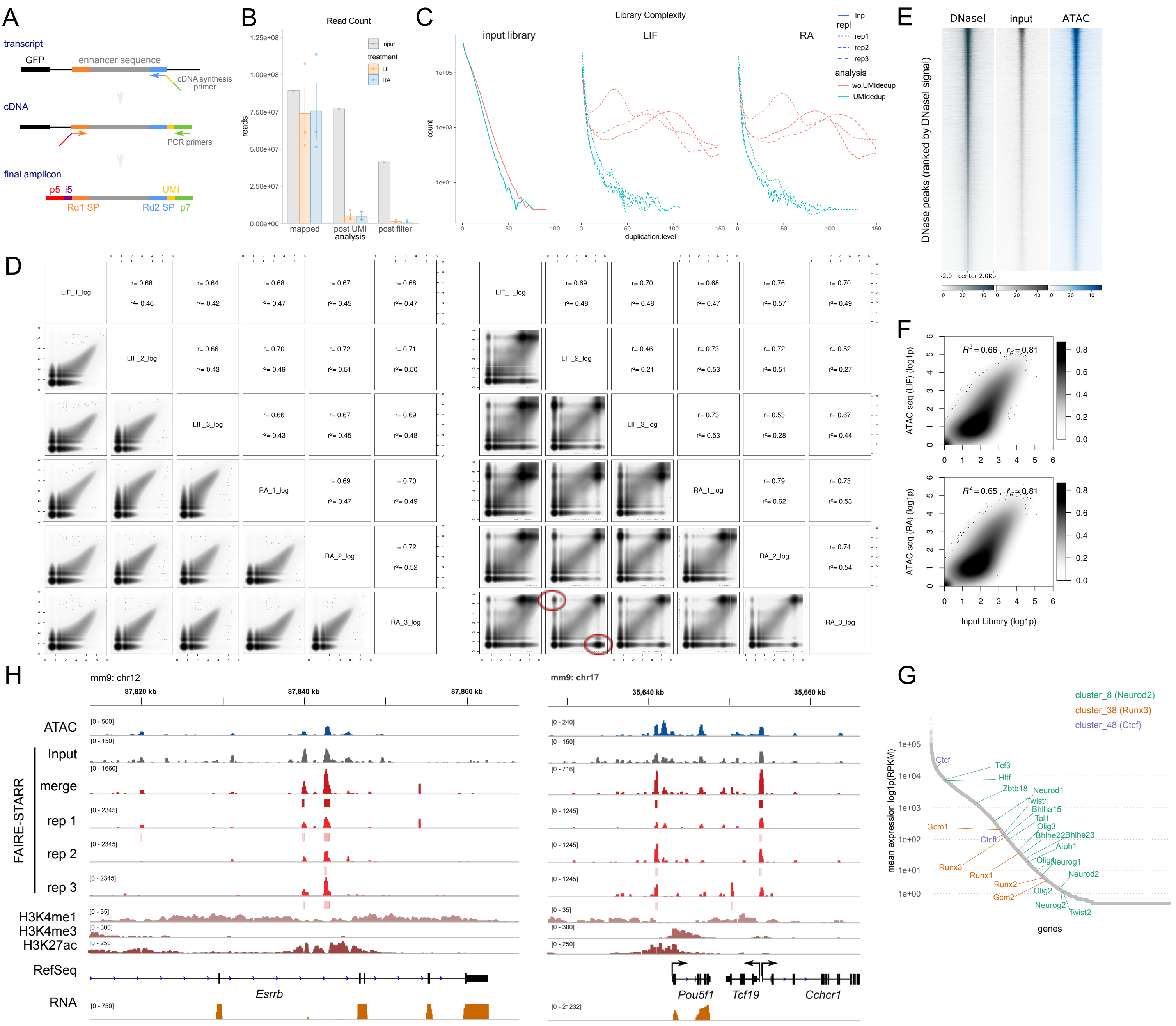

Figure S1. FAIRE-STARR-seq in mouse embryonic stem cells.

A) Schematic depiction of the FAIRE-STARR sequencing library preparation steps and final amplicon structure. Rd1/2 SP = Illumina Read1/2 Sequencing Primer, i5 = p5 index, UMI = unique molecular identifier (8 nucleotides). B) Read counts of the input compared to the FAIRE-STARR-seq libraries. Absolute read numbers were counted after sequencing, after UMI-aware deduplication, and after subsequent filtering for the input and the LIF or RA treated FAIRE-STARR libraries individually. The mean of three biological FAIRE-STARR-seq replicates (bars) as well as the counts of the individual replicates (points) are shown. C) Depiction of library complexities of the input library (Inp), LIF treated, and RA treated FAIRE-STARR libraries. Data with or without UMI-aware deduplication of reads is shown for the three individual FAIRE-STARR libraries (rep1-3). D) Correlation analysis of genome-wide read distribution, comparing the three individual biological replicates for FAIRE-STARR-seq after LIF or RA treatment after (left panels) or prior to (right panels) UMI-aware read deduplication. The genome was binned into 100 bp bins and log-transformed reads per bin are plotted. Pearson correlation (r) coefficient and r-square (r^2) of the log-transformed data for each comparison are shown. E) Heatmaps depicting normalized read distribution of DNase-seq, FAIRE-STARR input library, and ATAC-seq at the accessible regions based on the DNase-seq data. F) Analogous to Fig. 1C, correlation analysis of genome-wide read distribution comparing the input library to ATAC-seq data of LIF- or RA-treated mESCs. Normalized and log1p transformed reads per 10 kb genomic bin are shown. G) Mean expression (RPKM normalized) for all mESC genes in pluripotency. TF genes belonging to motif clusters 8, 28, and 48 are highlighted. H) IGV browser view of exemplary genomic regions illustrating the STARR activity from three individual biological replicates (rep1-3) and the merged signal. RPGC normalized signals for FAIRE-STARR replicates and input are shown.

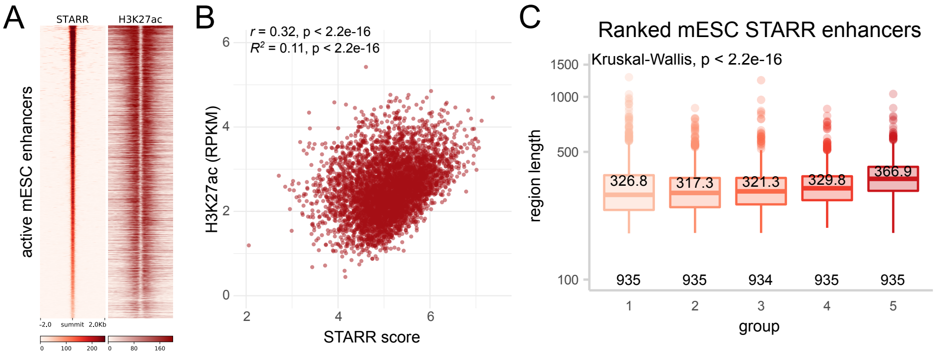

Figure S2. FAIRE-STARR-seq enables quantification of enhancer activity and activity level-associated sequence features.

A) Normalized FAIRE-STARR and H3K27ac signal distribution at FAIRE-STARR enhancers ranked by STARR activity. B) Correlation of STARR score and H3K27ac (sequencing depth and input normalized RPKM) signal at active enhancers. Pearson correlation coefficient (r) and coefficient of determination (R^2) are indicated. C) Average sequence length for each group of FAIRE-STARR enhancers (grouping as depicted in Fig. 2A). Boxplots depict the length-distribution of all genes per group, whiskers extend to 1.5 IQR. P-values were calculated by Kruskal-Wallis test for differences between all groups and group sizes are indicated directly above the x-axis.

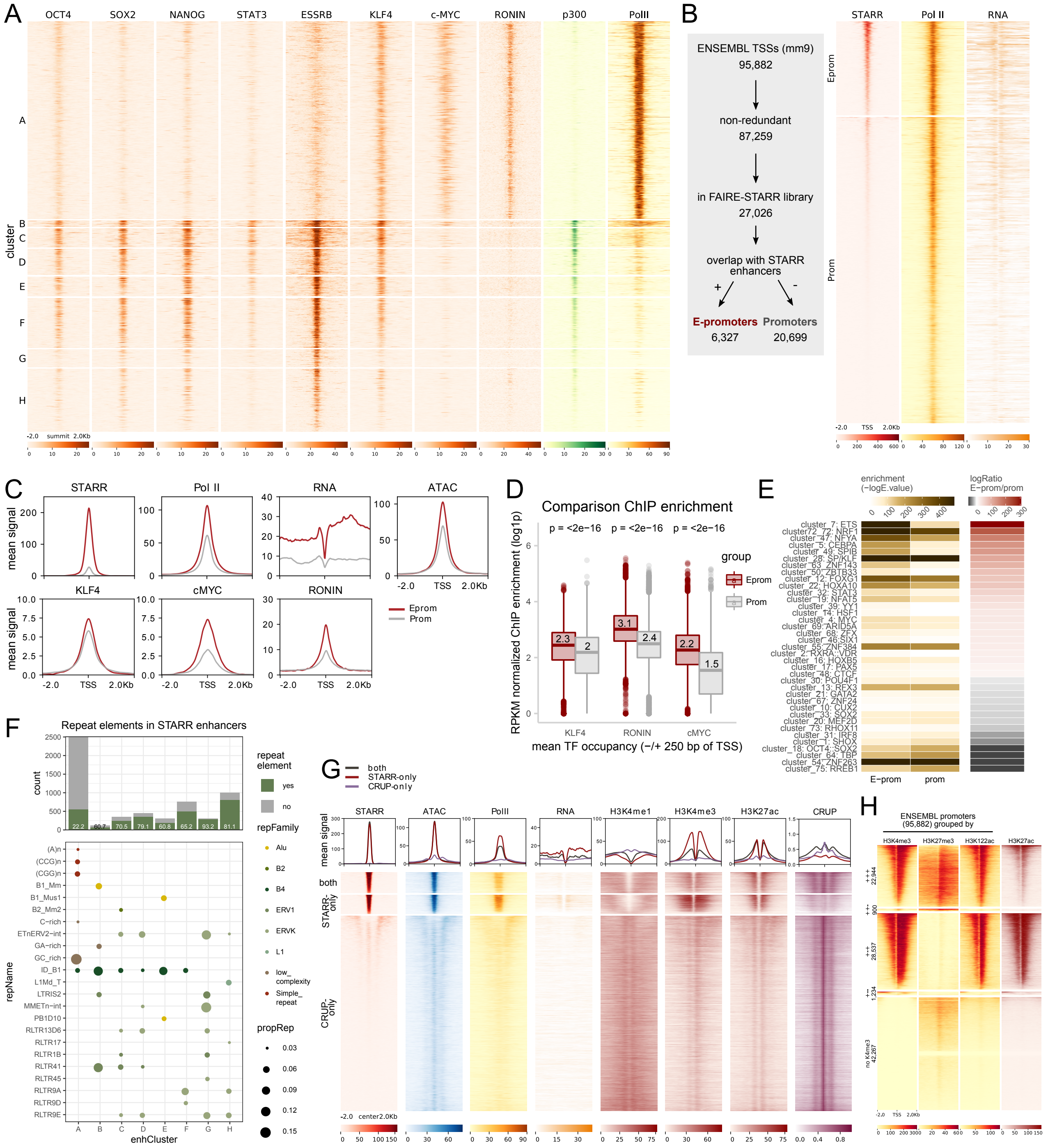

Figure S3. Functional mESC enhancers reside in different epigenomic environments.

A) Distribution of TFs as indicated, p300, and PolII at active FAIRE-STARR enhancers, clustered as depicted in Fig. 3A. B) TSSs (ENSEMBL annotation mm9) were filtered for redundancy and coverage in the FAIRE-STARR library and subsequently divided into those which do (E-promoters, Eprom) or do not (regular promoters, Prom) overlap with active FAIRE-STARR enhancers. The two groups were ranked by their STARR signal and PolII and RNA enrichment at these regions was plotted. C) Anchor plots showing the mean enrichment of signals as indicated at E-promoters and regular promoters (grouped as in B). D) Comparison of TF enrichment at E-promoters and promoters (-/+ 250 bp from TSSs). Boxplots depict the distribution of RPKM normalized ChIP signal (log1p transformed data) and p-values for unpaired Wilcoxon tests comparing enrichment for E-promoters and promoters. E) TF motif enrichment for E-promoters and (6327 randomly selected) promoters was performed with AME using the JASPAR 2018 clustered vertebrate motif database. Significance of enrichment (-log10E <= 1e-4) for differentially enriched (-log ratio >= 5) motifs are shown. F) Intersection of FAIRE-STARR enhancer clusters with elements from RepeatMasker (29) for the mm9 genome. The bar plot shows the absolute count for repeat elements per enhancer group and the percentage of all repeats per group are indicated. The lower panel shows the proportion of individual repeats, color-coded by repeat family, for each cluster. Only repeats which make up at least 3% of all repeats per cluster are shown. G) Mean enrichment (upper panels, anchor plots) and distribution (heatmaps) of STARR-, ATAC-, RNA-, selected HM ChIP-seq signals, as well as enhancer probability by CRUP prediction for enhancers identified only by FAIRE-STARR, only by CRUP or by both. H) Distribution of selected HMs at ENSEMBL promoters (95,882), which were grouped by overlap with significant enrichment (by peak calling) of H3K4me3, H3K27me3, and H3K122ac. +++: H3K4me3, K3K27me3, and H3K122ac positive. ++-: H3K4me3, K3K27me3, but no H3K122ac. +-+: H3K4me3 and H3K122ac positive, but no K3K27me3. +--: H3K4me3 positive, but no K3K27me3 or H3K122ac. No H3K4me3: Promoters without significant H3K4me3 enrichment. H3K27ac enrichment was plotted for the promoters grouped as described above.

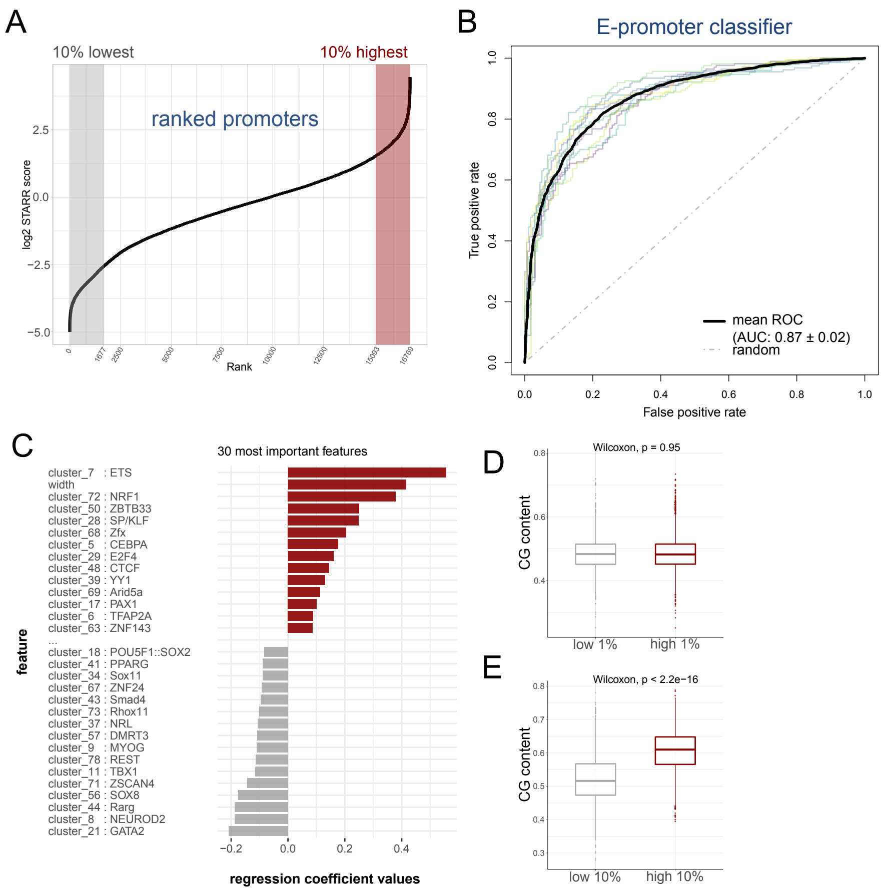

Figure S4: Sequence-based prediction of enhancers and E-promoters.

A) ENSEMBL promoters overlapping with FAIRE-STARR library regions (Fig. 4A) were ranked for their STARR score and the 10% highest and lowest ranking promoters were used for model building. B) Receiver operating characteristic (ROC) curve for E-promoter prediction model performance for each of the outer cross-validation folds and mean and standard deviation of the area under the ROC curve are shown. C) The 30 most predictive variables for the optimal model of E-promoter prediction and their coefficients are shown. Positive coefficients indicate a positive association with high STARR scores, while motifs with negative coefficients are associated with low-scoring elements. Comparison of CG content of high- and low-ranking D) enhancers and E) E-promoters. P-values for Wilcoxon test are depicted.

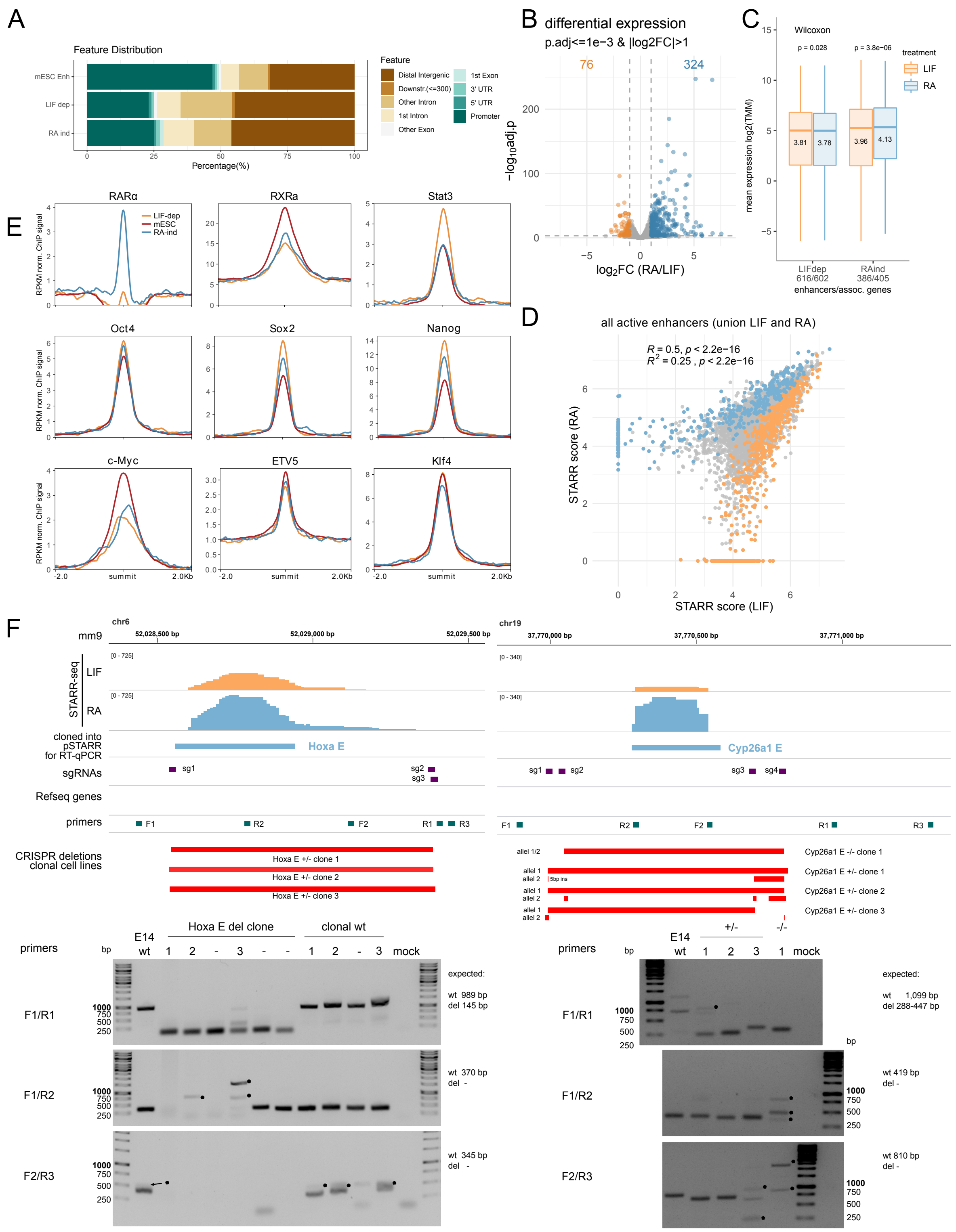

Figure S5: Differentiation-associated changes in enhancer activity.

A) Genomic distribution of active enhancers in mESCs, of LIF-dependent and RA-inducible enhancers with respect to annotated Refseq genes. Promoters were defined as the regions 1 kb upstream of a TSS. B) Differential gene expression comparing E14 cells treated for 4 h with either LIF or RA using DESeq2. Significantly up- (blue) and down-regulated (orange) genes and cut-offs are indicated. C) Genes were paired with enhancers by distance using GREAT and TMM-normalized gene expression counts per enhancer group and treatment are shown. P-values from paired Wilcoxon tests are shown. D) Correlation of STARR score at all active enhancers (union of active enhancers identified from LIF and RA treated cells) comparing LIF and RA treatment. Colored dots display significant differentially active enhancers called by our analysis pipeline (orange: LIF-dependent, blue: RA-induced enhancers). Pearson correlation coefficient (R) and coefficient of determination (R^2) are indicated. E) Mean normalized enrichment of TFs as indicated at LIF-dependent, RA-inducible, and active mESC STARR enhancers. F) Genotyping of E14 enhancer deletion CRISPR/Cas9 clones. Upper panels depict the targeted genomic regions (mm9) and FAIRE-STARR-seq signals (LIF or RA treated). Genomic locations of regions cloned into pSTARR for RT-qPCR (blue, Fig. 5E and F), guide RNAs for targeting Cas9 (sgRNAs, purple), primers (green) used for genotyping PCRs, and detected deletions (red) of the individual clones are depicted. Lower panels show genotyping PCR results for genomic DNA recovered from individual clones or parental line (E14 wt) using primer pairs as indicated (locations shown in the upper panel). Asterix mark unspecific PCR bands. Expected PCR amplicon sizes for deletion (del) or wild type (wt) clones are indicated.

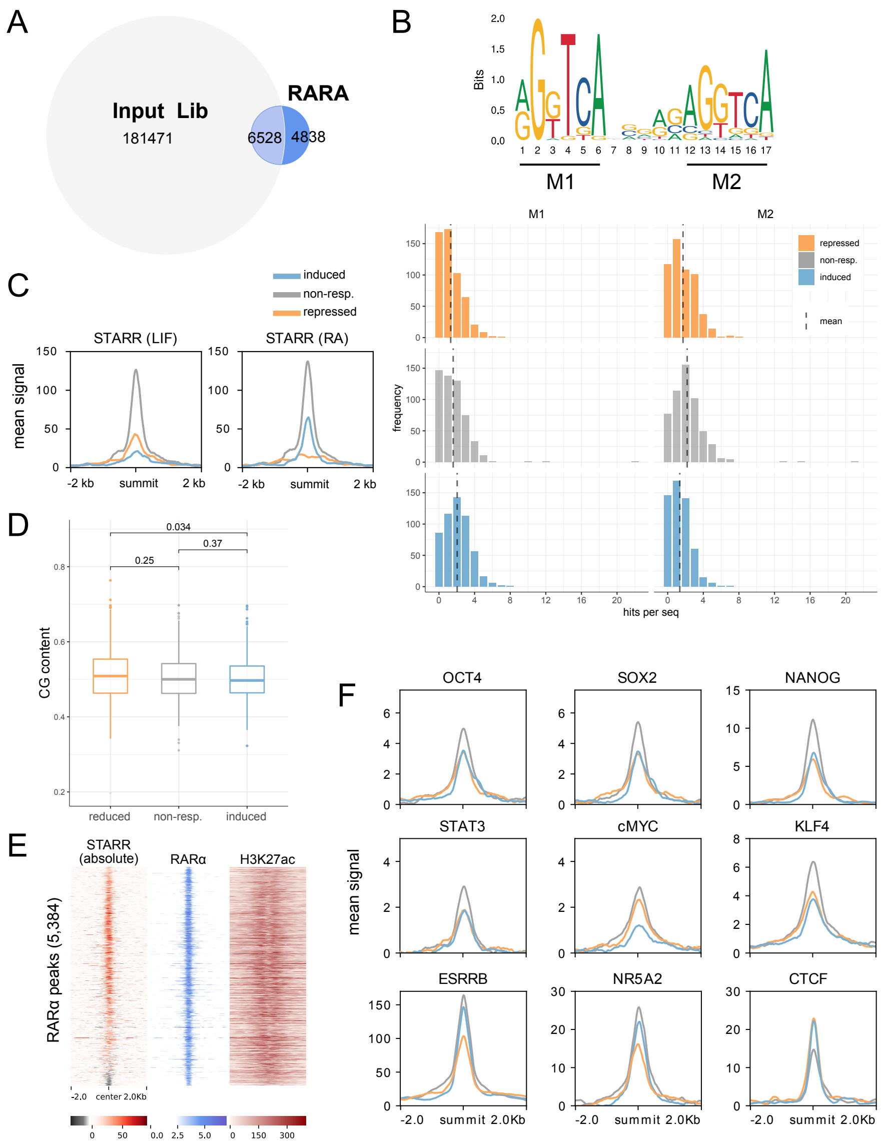

Figure S6: Identification of enhancer activity-associated features of RARα binding sites.

A) Intersection of FAIRE-STARR-seq input library and RARα-occupied sites. 6,528 RARα sites covered by our FAIRE-STARR-seq input library were used for further analysis. B) Frequencies of the RARα::RXRα consensus motif (MA0159.1) repeat half-sites (M1 and M2, upper panel) at repressed, non-responsive, and induced RARα-occupied sites. C) Absolute FAIRE-STARR activity at repressed, non-responsive, and induced RARα-occupied sites in LIF or RA treated cells. D) CG content distribution at repressed, non-responsive, and induced RARα-occupied sites. P-values were derived from Wilcoxon tests. E) Heatmap representing distribution of FAIRE-STARR signal, RARα and H3K27ac enrichment at RARα-occupied sites which were ranked by their logSTARR score (RA/LIF). F) Average enrichment of selected TFs at induced (blue), non-responding (gray), or repressed (orange) RARα-occupied sites. All ChIP-seq data were derived from pluripotent mESCs without RA treatment.

References

1. Arnold,C.D., Gerlach,D., Stelzer,C., Boryn,L.M., Rath,M. and Stark,A. (2013) Genome-Wide Quantitative Enhancer Activity Maps Identified by STARR-seq. *Science (80-.).*, **339**, 1074–1077.

2. Klemm,S.L., Shipony,Z. and Greenleaf,W.J. (2019) Chromatin accessibility and the regulatory epigenome. *Nat. Rev. Genet.*, **20**, 207–220.

3. Schöne,S., Bothe,M., Einfeldt,E., Borschiwer,M., Benner,P., Vingron,M., Thomas-Chollier,M. and Meijsing,S.H. (2018) Synthetic STARR-seq reveals how DNA shape and sequence modulate transcriptional output and noise. *PLOS Genet.*, **14**, e1007793.

4. Muerdter,F., Boryn,Ł.M., Woodfin,A.R., Neumayr,C., Rath,M., Zabidi,M.A., Pagani,M., Haberle,V., Kazmar,T., Catarino,R.R., *et al.* (2018) Resolving systematic errors in widely used enhancer activity assays in human cells. *Nat. Methods*, **15**, 141–149.

5. Wang,R., Wang,J., Paul,A.M., Acharya,D., Bai,F., Huang,F. and Guo,Y.L. (2013) Mouse embryonic stem cells are deficient in type i interferon expression in response to viral infections and double-stranded RNA. *J. Biol. Chem.*, **288**, 15926–15936.

6. Glaser,L.V., Rieger,S., Thumann,S., Beer,S., Kuklik-Roos,C., Martin,D.E., Maier,K.C., Harth-Hertle,M.L., Grüning,B., Backofen,R., *et al.* (2017) EBF1 binds to EBNA2 and promotes the assembly of EBNA2 chromatin complexes in B cells. *PLoS Pathog.*, **13**.

7. Langmead,B. and Salzberg,S.L. (2012) Fast gapped-read alignment with Bowtie 2. *Nat. Methods*, 10.1038/nmeth.1923.

8. Smith,T., Heger,A. and Sudbery,I. (2017) UMI-tools: Modeling sequencing errors in Unique Molecular Identifiers to improve quantification accuracy. *Genome Res.*, 10.1101/gr.209601.116.

9. Li,H., Handsaker,B., Wysoker,A., Fennell,T., Ruan,J., Homer,N., Marth,G., Abecasis,G. and Durbin,R. (2009) The Sequence Alignment/Map format and SAMtools. *Bioinformatics*, 10.1093/bioinformatics/btp352.

10. Zhang,Y., Liu,T., Meyer,C.A., Eeckhoute,J., Johnson,D.S., Bernstein,B.E., Nussbaum,C., Myers,R.M., Brown,M., Li,W., *et al.* (2008) Model-based analysis of ChIP-Seq (MACS). *Genome Biol.*, 10.1186/gb-2008-9-9-r137.

11. Ramírez,F., Ryan,D.P., Grüning,B., Bhardwaj,V., Kilpert,F., Richter,A.S., Heyne,S., Dündar,F. and Manke,T. (2016) deepTools2: a next generation web server for deep-sequencing data analysis. *Nucleic Acids Res.*, 10.1093/nar/gkw257.

12. Yu,G., Wang,L.-G. and He,Q.-Y. (2015) ChIPseeker: an R/Bioconductor package for ChIP peak annotation, comparison and visualization. *Bioinformatics*, **31**, 2382–2383.

13. Ramisch,A., Heinrich,V., Glaser,L. V., Fuchs,A., Yang,X., Benner,P., Schöpflin,R., Li,N., Kinkley,S., Römer-Hillmann,A., *et al.* (2019) CRUP: a comprehensive framework to predict condition-specific regulatory units. *Genome Biol.*, **20**, 227.

14. R Core Team (2017) R: A language and environment for statistical computing.

15. Dunham,I., Kundaje,A., Aldred,S.F., Collins,P.J., Davis,C.A., Doyle,F., Epstein,C.B., Frietze,S., Harrow,J., Kaul,R., *et al.* (2012) An integrated encyclopedia of DNA elements in the human genome. *Nature*, **489**, 57–74.

16. Zhao,H., Sun,Z., Wang,J., Huang,H., Kocher,J.-P. and Wang,L. (2014) CrossMap: a versatile tool for coordinate conversion between genome assemblies. *Bioinformatics*, **30**, 1006–1007.

17. Dobin,A., Davis,C.A., Schlesinger,F., Drenkow,J., Zaleski,C., Jha,S., Batut,P., Chaisson,M. and Gingeras,T.R. (2013) STAR: Ultrafast universal RNA-seq aligner. *Bioinformatics*, **29**, 15–21.

18. Liao,Y., Smyth,G.K. and Shi,W. (2014) FeatureCounts: An efficient general purpose program for assigning sequence reads to genomic features. *Bioinformatics*, **30**, 923–930.

19. Love,M.I., Huber,W. and Anders,S. (2014) Moderated estimation of fold change and dispersion for RNA-seq data with DESeq2. *Genome Biol.*, **15**, 550.

20. Robinson,M.D. and Oshlack,A. (2010) A scaling normalization method for differential expression analysis of RNA-seq data. *Genome Biol.*, **11**, R25.

21. Robinson,M.D., McCarthy,D.J. and Smyth,G.K. (2010) edgeR: a Bioconductor package for differential expression analysis of digital gene expression data. *Bioinformatics*, **26**, 139–140.

22. McLeay,R.C. and Bailey,T.L. (2010) Motif Enrichment Analysis: A unified framework and an evaluation on ChIP data. *BMC Bioinformatics*, **11**, 165.

23. Khan,A., Fornes,O., Stigliani,A., Gheorghe,M., Castro-Mondragon,J.A., Van Der Lee,R., Bessy,A., Chèneby,J.,

Kulkarni,S.R., Tan,G., *et al.* (2018) JASPAR 2018: Update of the open-access database of transcription factor binding profiles and its web framework. *Nucleic Acids Res.*, **46**, D260–D266.

24. Wickham,H. (2009) ggplot2: Elegant Graphics for Data Analysis.

25. Turatsinze,J.V., Thomas-Chollier,M., Defrance,M. and van Helden,J. (2008) Using RSAT to scan genome sequences for transcription factor binding sites and cis-regulatory modules. *Nat. Protoc.*, **3**, 1578–1588.

26. McLean,C.Y., Bristor,D., Hiller,M., Clarke,S.L., Schaar,B.T., Lowe,C.B., Wenger,A.M. and Bejerano,G. (2010) GREAT improves functional interpretation of cis-regulatory regions. *Nat. Biotechnol.*, **28**, 495–501.

27. W,K. and M,V. (2017) An improved compound Poisson model for the number of motif hits in DNA sequences. *Bioinformatics*, **33**, 3929–3937.

28. Zou,H. and Hastie,T. (2005) Regularization and variable selection via the elastic net.

29. Friedman,J., Hastie,T. and Tibshirani,R. (2010) Regularization paths for generalized linear models via coordinate descent. *J. Stat. Softw.*, **33**, 1–22.
