# Supplementary material for: Assessing genome-wide dynamic changes in enhancer activity during early mESC differentiation by FAIRE-STARR-seq
Source: Nucleic Acids Res. 2021 Nov 24;49(21):12178–95. doi: 10.1093/nar/gkab1100 (PMC8643627; doi:10.1093/nar/gkab1100)
Supplement: gkab1100_Supplemental_Files [file gkab1100_supplemental_files.zip › Supplementary_Tables.docx]

**Supplementary Tables**

**Supplementary Table 1: DNA sequences of STARR constructs, gRNAs, and primers.**

**Supplementary Table 2: Sources of publicly available NGS data reanalyzed for this study.**

**Supplementary Table 3: Genotypes of genomic enhancer deletion mESC clones.**

**Supplementary Table 4: JASPAR 2018 clustered vertebrate motifs.**
